# Optimal Target Assignment with Seamless Handovers for Networked Radars

**DOI:** 10.3390/s19204555

**Published:** 2019-10-19

**Authors:** Juhyung Kim, Doo-Hyun Cho, Woo-Cheol Lee, Soon-Seo Park, Han-Lim Choi

**Affiliations:** 1 Department of Aerospace Engineering, Korea Advanced Institute of Science and Technology, Daejeon 34141, Korea; jhkim@lics.kaist.ac.kr (J.K.); wclee@lics.kaist.ac.kr (W.-C.L.); sspark@lics.kaist.ac.kr (S.-S.P.); 2 Mechatronics R&D Center, Samsung Electronics, Hwaseong 18448, Korea; dhcho@lics.kaist.ac.kr; 3 Department of Aerospace Engineering & KI for Robotics, Korea Advanced Institute of Science and Technology, Daejeon 34141, Korea

**Keywords:** target handover, seamless multi-target tracking, radar network systems, optimal scheduling, situational awareness

## Abstract

This paper proposes a binary linear programming formulation for multiple target assignment of a radar network and demonstrates its applicability to obtain optimal solutions using an off-the-shelf mixed-integer linear programming solver. The goal of radar resource scheduling in this paper is to assign the maximum number of targets by handing over targets between networked radar systems to overcome physical limitations such as the detection range and simultaneous tracking capability of each radar. To achieve this, time windows are generated considering the relation between each radar and target considering incoming target information. Numerical experiments using a local-scale simulation were performed to verify the functionality of the formulation and a sensitivity analysis was conducted to identify the trend of the results with respect to several parameters. Additional experiments performed for a large-scale (battlefield) scenario confirmed that the proposed formulation is valid and applicable for hundreds of targets and corresponding radar network systems composed of five distributed radars. The performance of the scheduling solutions using the proposed formulation was better than that of the general greedy algorithm as a heuristic approach in terms of objective value as well as the number of handovers.

## 1. Introduction

The rapid development of computer and communications technologies since the 1980s led to a new doctrine in the military field of the United States under the name of Network-Centric Warfare (NCW) between the late 1990s and early 2000s [1,2]. The introduction of this concept enabled faster and better decision making on the battlefield, based on integrated situational awareness through the convergence and processing of information gathered by the networked sensors. Platforms in charge of attack or defense became able to respond quickly to enemy threats, using the integrated sensors and shooters, according to these decisions. The typical example of these networked systems-of-systems in the military field is the ballistic missile defense system [3]. The ballistic missile defense systems consist of precise surveillance radar networks with various types of platforms such as early warning radar and local air defense radar, and their combined intercept weapon systems [4].

One of the most dangerous enemy provocations that can be expected is simultaneous multiple ballistic missile attacks. To protect against such a situation in a timely manner, and to minimize damage, a very strictly constructed air defense system is necessary, one that can take into account precise information (missile type, trajectory, aim point, etc.) about the enemy missiles. In order for such an air defense system to perform properly, all sensing and intercept systems of the entire battlefield must systemically exchange information, and efficient decision-making should be performed based on that information. The typical processes for eliminating the ballistic missile threat are the target detection and identification, tracking and trajectory estimation, target evaluation, weapon–target assignment (WTA), and effective decision making, considering the flight phase of the ballistic missiles [5].

This paper proposes a novel sensor scheduling method to integrate heterogeneous sensor systems for a future battlefield where various type of sensors and intercept systems with diverse capabilities coexist. In particular, the main contribution of this paper is to provide the concept of seamless tracking that utilizes target handover between radars to have better situational awareness by using binary Mixed Integer Linear Programming (MILP) formulation. For simulations similar to real-world situations, it is assumed that early warning radar (EWR) catches and disseminates the entire battlefield situation, including target information. The time windows are generated considering the relative positions and velocities between radars and incoming targets. The time window thus generated represents the time period in which each radar can detect and track a target. Seamless tracking is a concept that allows continuous tracking by handing over targets to different radars that have not been assigned yet when encountering the limits of individual radars. In this study, a binary linear programming formulation was mainly used as a scheduling method to assign the tracking period to the appropriate time windows. To verify the effectiveness of the solution performed by off-the-shelf MILP solver, an additional heuristic approach was also implemented in the simulation experiment. For heuristic approach, we used and named First-In First-Out (FIFO) greedy algorithm that can implement a target handover situation. Many different MILP scheduling studies use the greedy algorithm together to compare performance [6,7]. Conversely, MILP formulations can sometimes be used to compare the performance of specially designed greedy algorithms [8,9,10].

The concept of how to operate multiple radar resources in a networked fashion is well documented in a paper by Green et al. [11]. Narykov et al. developed a sensor management algorithm for target tracking that uses multiple phased array radars to minimize the sensor system load [12]. Lian proposed a sensor selection optimization algorithm that can track multiple targets using a decentralized large-scale network within a labeled random finite set (RFS) framework [13]. Closer to the topic of this paper, Fu et al. proposed distributed sensor allocation for tracking multiple targets in wirelessly connected sensor networks; to improve the tracking performance, they solved the sensor fusion problem and the allocation optimization problem for the sensor and the whole target [14]. Yan introduced a method to optimize radar assignment for multiple targets, taking into account the limited time resources of each radar in the situation of detecting/tracking multiple targets with multiple networked multi-function phased array radars. This was a way to maintain the detection performance of the entire radar network even in overload situations that exceeded the tracking capability of individual radars [15]. Sherwani and Griffiths proposed a method to control the tracking parameters in order to construct an information sharing system that integrates multi-function radar networks, which are inherently limited in resource management, into one system [16]. Severson and Paley optimized radar resource management for ballistic missile reconnaissance and tracking through a decentralized consensus-based approach. Through this approach, each radar could determine their preferred radar–target allocation by balancing the radar load and minimizing the use of total radar [17]. They later solved the problem of optimal sensor coordination and tracking allocation so that multiple shipboard radars could integrate so to expand their search area and the number of tracking targets [18]. Regarding the radar scheduling using the concept of time windows, Chaolong et al. [19], Jang and Choi [20], Duan et al. [21], and Qiang et al. [22] introduced time window into the multi function phased array radar’s task scheduling problems.

However, still the aforementioned studies do not contain a methodology for mathematical optimization considering the handover. The research most closely related to target handover for seamless tracking are studies on track to track correlation between the radar track and onboard IR track picture [23]. According to Lewis and Tabaczynski [24], handover technology was first achieved in 2003 between radars, and RF-to-RF and RF-to-IR handover was achieved in 2005. As the data association and sensor fusion technology developed, target handover technique is also evolving. On this basis, we are dealing with the long time-frame seamless tracking for multiple targets.

The rest of the paper is organized as follows. Section 2 introduces concepts for this study and describes the problem in detail. In Section 3, the problem is formally stated and explained in detail. Numerical simulation results are provided and discussed in Section 4. Finally, Section 5 discusses the conclusions of this study.

## 2. Preliminaries

### 2.1. Mission Overview

The problem of radar network resource management for ballistic missile defense is to deal with the schedule assignments of individual radars to precisely track the target. In this paper, we concentrate on the resource management problem of local radars that can perform precise target tracking, assuming that there is an EWR systems that can observe the whole battlefield situation. The objective function of radar resource assignment in a multi-target multi-radar situation should consider: (1) target priority for each radar; (2) continuity of target tracking; and (3) maximization of the number of tracked targets for the entire networked radar systems.

**Figure 1:**
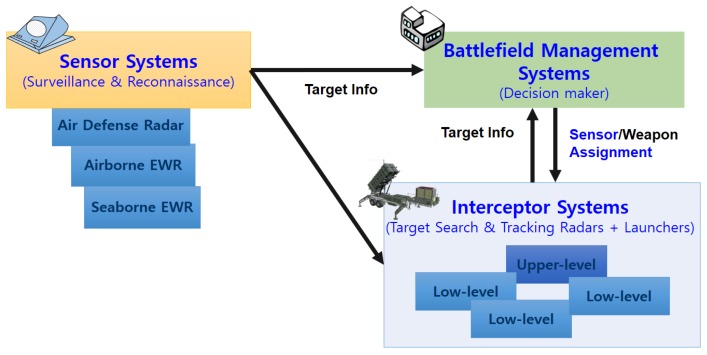
Schematic of system related to scheduling problem.

The decision maker in Figure 1 performs local radar resource management. Prior knowledge (predicted target trajectory) for resource management can be obtained through the EWRs in sensor systems, which can observe a relatively large area compared to local radar.

### 2.2. Key Notions in Scheduling

#### 2.2.1. Radar

The radar parameters reflected in the resource management algorithm are the maximum number of targets that can be tracked simultaneously and the coverage of the radar. Radars are limited in the number of targets that can be tracked to the maximum according to the characteristics of each radar, and the maximum number of targets being tracked can be estimated according to the minimum tracking performance requirement. In this paper, to deal with large scale problems, the maximum number of targets per radar is arbitrarily assumed. If multiple radars with limited coverage are placed in different locations with different azimuth angles, they will have different time windows for the same target. For multiple target situations, the time window becomes more complex, and this makes the multiple target multiple radar resource management problem difficult. In this paper, the radar coverage is determined by radar position, tilt angle, azimuth direction range, altitude angular range, and distance direction range.

#### 2.2.2. Target Priority

The target importance needs to be assessed using a priority-based metric that reflects the relative distance and remaining time between the radar and the target. Therefore, even if the same target is tracked by two or more radars, the target importance is different for each radar.

In this paper, since it is difficult to quantify the degree of threat according to the type of target, the target importance is calculated using the time remaining until the target hits the surface and the distance between the radar and the target. Here, the impact time of the target reflects the urgency to engage the target. Thus, it sets a higher priority when the remaining time becomes smaller. For a fast target, the remaining time will decrease very quickly, and thus the increasing rate of the tracking value over time will be higher than those of other targets. The distance between the target and the radar is a factor that reflects the Signal-to-Noise Ratio (SNR) and hence the expected tracking performance. Therefore, the target priority used in this study reflects the expected performance and urgency. The tracking value ($v_{t}$), determined by remaining time to impact (τ) and the distance from the radar (dist), is calculated as follows [25].

(1) $$v_{t}=\left(1-\frac{1}{1+e^{-\left(\tau -\tau_{0}\right)/\alpha_{\tau }}}\right)+\left(1-\frac{\beta_{dist}}{1+e^{-\left(dist-dist_{0}\right)/\alpha_{dist}}}\right)$$

where $\tau_{0}$, $\alpha_{\tau }$, $dist_{0}$, $\alpha_{dist}$, and $\beta_{dist}$ are parameters to determine the shape of the sigmoid function. $\tau_{0}=100$, $\alpha_{\tau }=15$, $dist_{0}=500$, $\alpha_{dist}=100$, and $\beta_{dist}=0.8$ are used in this work.

Equation (1) decreases non-linearly (sigmoid) as the distance increases and reflects the change in average tracking performance according to SNR when the target is tracked in a single radar with a fixed resource.

#### 2.2.3. Ballistic Target

The ballistic missile model is simulated including phases of boost, free-flight, and reentry as described in [26]. The acceleration acting on the ballistic target in each phase is expressed as follows.

(2) $$\begin{array}{cc} \mathrm{Boost}\;\mathrm{phase}:\;a & =a_{thrust}+a_{drag}+a_{gravity}\\ \mathrm{Free}-\mathrm{flight}\;\mathrm{phase}:\;a & =a_{gravity}\\ \mathrm{reentry}\;\mathrm{phase}:\;a & =a_{drag}+a_{gravity} \end{array}$$

where

(3) $$\begin{array}{cc} a_{thrust} & =-\frac{T}{m}u_{T}\\ a_{gravity} & =-\frac{\mu }{{\left\|x\right\|}^{3}}x\\ a_{drag} & =-\frac{\rho (h)\|v\|}{2\beta }v \end{array}$$

**Figure 2:**
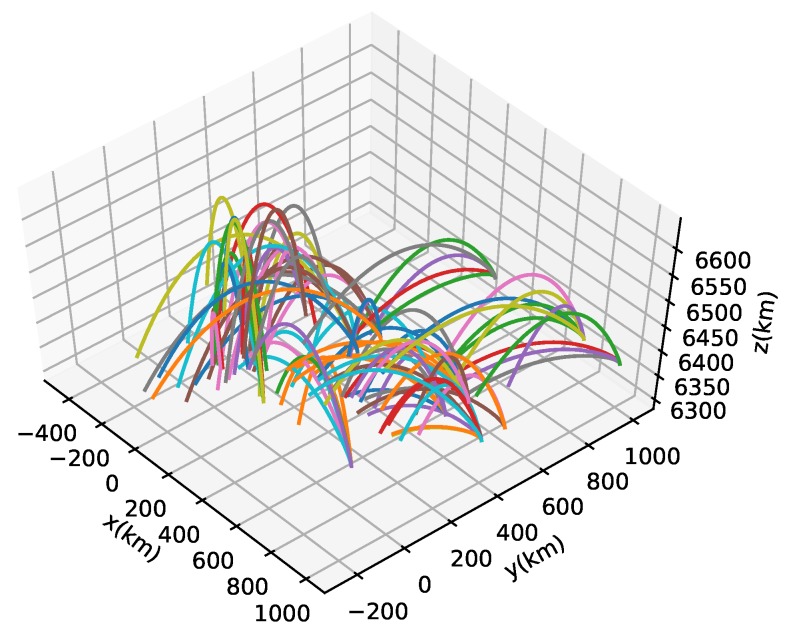
Randomly generated sample trajectories of ballistic targets.

Here, acceleration regarding Coriolis force can be added according to the coordinate system [26]. In Equation (3), **T** stands for the thrust magnitude, *m* stands for target mass, $u_{T}$ stands for the unit vector which indicates thrust direction, μ stands for the Earth’s gravitational constant, **x** stands for the vector from the Earth center to the target, ρ stands for the air density, *h* denotes target altitude, β stands for the ballistic coefficient, and **v** denotes the target velocity vector. Based on this, the trajectories of the ballistic targets in the Earth-Centered Earth-Fixed (ECEF) coordinate systems were generated, as can be seen in Figure 2.

#### 2.2.4. Time Window

Each radar has a time window if the target trajectory for each target is within the coverage of the radar. The time window consists of the release (start) time and the due (end) time; the times at which the target enters and leaves the coverage of the radar are set as the release time and the due time of the time window, respectively. If Radar *r* has a time window for Target *t* and tracking is performed, the observation start time and the observation progress time are determined, and the constraint of the problem is specified so that tracking is performed only within the corresponding time window.

#### 2.2.5. Handover

To update the state of the target for as long as possible, target needs to be tracked through multiple radars. Suppose Radar $r_{1}$ and $r_{2}$ are close to each other and lie in the same direction. In the scenario in which Target *t* enters coverage of $r_{1}$, enters coverage of $r_{2}$, leaves coverage of $r_{1}$ first, and then leaves coverage of $r_{2}$, the scheduling that $r_{1}$ observes first and that $r_{2}$ then observes can be thought proper. We define “Radar $r_{1}$ hand over target to Radar $r_{2}$” for the situation in which Radar $r_{1}$ tracks the target with Radar $r_{2}$ until stable measurement can be obtained after Radar $r_{2}$ starts target tracking.

### 2.3. Toy Model Implementation

**Figure 3:**
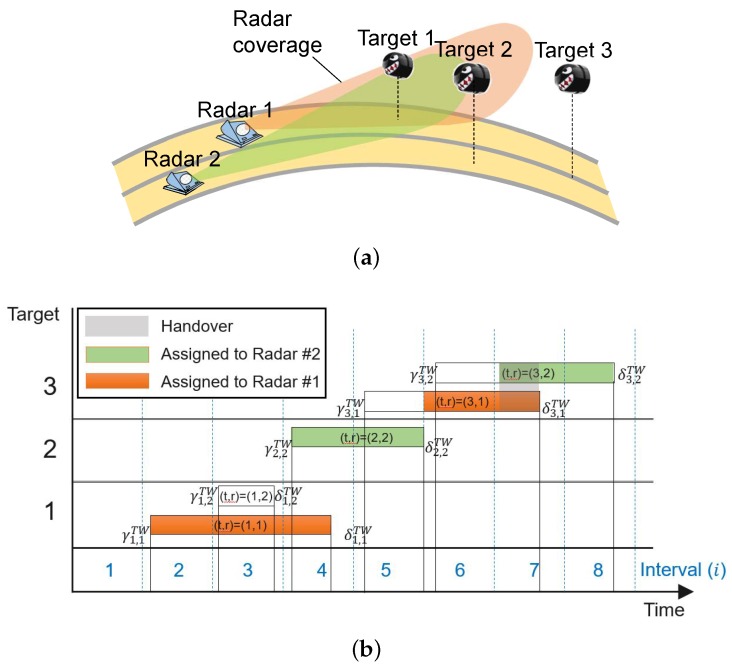
Conceptual diagram for sensor assignment considering target handover (refer to Table 1 for definitions of the symbols). (**a**) Physical circumstance description. (**b**) Description of time windows and handover procedure for seamless tracking.

A toy version of the scheduling problem with three targets and two radars is shown in Figure 3. Given knowledge about each target’s trajectory and a set of radars, time windows for each target–radar pair can be calculated to ensure the maximum radar coverage. The time windows can be calculated simply by checking whether a target is inside the coverage of the radar or not; the coverage and assignments of each radar are colored differently depending on the radar. Suppose that each radar can track only a single target at a given time ($n^{\mathrm{capa}}=1$), and the quality of measurement from a single radar is sufficiently high that simultaneous tracking by multiple radars is not needed in the given instance. The assignment results for the situation in Figure 3a are obtained as in Figure 3b. For Target $t_{1}$, since Time Window $TW_{1,2}$ includes Time Window $TW_{1,1}$, only Radar $r_{2}$ tracks the target. For Target $t_{2}$, $r_{2}$ tracks it because the time window exists only for $r_{1}$. For $t_{3}$, $r_{1}$ tracks $t_{3}$ first because Time Window $TW_{3,1}$ starts before Time Window $TW_{3,2}$, and then $r_{1}$ hands over the target to $r_{2}$ at Interval 7. For stable tracking, both radars $r_{1}$ and $r_{2}$ simultaneously measure the target during the handover period in Interval 7. The planning horizon is divided into intervals of equal length for checking tracking status. Let us look at the results for Intervals 5 and 6 in Figure 3b. Because, from the assumption of the problem, each radar can track only a single target, the assignment in Time Window $TW_{3,1}$ starts from the release time of Interval 6, $\gamma_{6}^{Int}$, rather than the release time of $TW_{3,1}$, $\gamma_{3,1}^{TW}$, and the time interval corresponding to Interval 5 of Time Window $TW_{3,1}$ is excluded. The assignment to Time Window $TW_{3,1}$ continues until $\delta_{3,1}^{TW}$. After the handover from $r_{1}$ to $r_{2}$, $r_{2}$ tracks $t_{3}$ until Time Window $TW_{3,2}$ is finished.

### 2.4. Assumptions

Before embodying the problem, several assumptions must be made in order to implement seamless handover between multiple radars for multiple targets.

- First, communication between the radars is fast enough to ensure appropriate information sharing. Communication connections using satellites or terrestrial optical cables should be a prerequisite.
- Second, since numerous researches have been conducted on sensor fusion and data association techniques for the handover of ballistic target information [23,24,27,28,29,30], it is regarded that the targets are handed over smoothly, and filtering problems related to target processing and sensor fusion that occurs are not covered in this study. The methodological and technical problems that may arise in the process of handing over targets between radars are not discussed. Please note that there is an early warning radar (EWR) featuring handover capability has recently been introduced in the market [31].
- Third, it is assumed that ballistic missile information is provided by EWR so that the time window for each missile is within the entire mission planning horizon. In addition, the EWR is equipped with a target separation and data association capability in the ground-to-air-level clutter environment.

## 3. Problem Formulation

Scheduling for the general multi-target and multi-radar model is formulated as the following equations. The parameters and decision variables for the objective function and for the constraints are described in Table 1 and Table 2.

The objective function is the sum of target–radar–interval assignment $\theta_{t,r,i}$, tracking duration $\tau_{t,r}^{p}$, and target assignment $x_{t}$ minus target–radar assignment $x_{t,r}$ with appropriate weight values for each term in the above formulation. The terms of the objective function have the following roles: the first term identifies the importance of the target–radar pair over time, the second term maximizes the tracking duration of the whole assignment, the third maximizes the number of targets to track, and the last one minimizes the number of handovers between different radars.

Maximize

(4) $$\begin{array}{c} c_{1}\sum_{\theta \in \Theta }w_{t,r,i}\theta_{t,r,i}+c_{2}\sum_{t\in T,r\in R}w_{t}\tau_{t,r}^{p}+c_{3}\sum_{t\in T}w_{t}x_{t}-c_{4}\sum_{t\in T,r\in R}w_{t}x_{t,r} \end{array}$$

subject to

(5) $$x_{t}=\max\left\{x_{t,1},\ldots ,x_{t,n^{R}}\right\}\;\;\forall t\in T$$

(6a) $$\begin{array}{ccc} \tau_{t,r}^{p} & \leq Mx_{t,r} & \;\forall t\in T,r\in R \end{array}$$

(6b) $$\begin{array}{ccc} \tau^{p,min}x_{t,r} & \leq \tau_{t,r}^{p} & \;\forall t\in T,r\in R \end{array}$$

(7a) $$\begin{array}{ccc} \gamma_{t,r}^{TW} & \leq \tau_{t,r}^{s} & \;\forall t\in T,r\in R \end{array}$$

(7b) $$\begin{array}{ccc} \tau_{t,r}^{s}+\tau_{t,r}^{p} & \leq \delta_{t,r}^{TW} & \;\forall t\in T,r\in R \end{array}$$

(8a) $$\begin{array}{ccc} \delta_{i}^{Int}-\tau_{t,r}^{s} & \leq M\theta_{t,r,i}^{start} & \;\forall t\in T,r\in R,i\in I \end{array}$$

(8b) $$\begin{array}{ccc} \tau_{t,r}^{s}+\tau_{t,r}^{p}-\gamma_{i}^{Int} & \leq M\theta_{t,r,i}^{end} & \;\forall t\in T,r\in R,i\in I \end{array}$$

(8c) $$\begin{array}{ccc} \theta_{t,r,i} & \leq x_{t,r} & \;\forall t\in T,r\in R,i\in I \end{array}$$

(8d) $$\begin{array}{ccc} \theta_{t,r,i} & \leq \theta_{t,r,i}^{start} & \;\forall t\in T,r\in R,i\in I \end{array}$$

(8e) $$\begin{array}{ccc} \theta_{t,r,i} & \leq \theta_{t,r,i}^{end} & \;\forall t\in T,r\in R,i\in I \end{array}$$

(8f) $$\begin{array}{ccc} \left(\begin{array}{c} x_{t,r}+\theta_{t,r,i}^{start}+\theta_{t,r,i}^{end}-2 \end{array}\right) & \leq \theta_{t,r,i} & \;\forall t\in T,r\in R,i\in I \end{array}$$

(9) $$\begin{array}{ccc} \sum_{r\in R}\theta_{t,r,i} & \leq 2 & \;\forall t\in T,i\in I \end{array}$$

(10) $$\begin{array}{ccc} \sum_{t\in T}\theta_{t,r,i} & \leq n^{\mathrm{capa}} & \;\forall r\in R,i\in I \end{array}$$

(11a) $$\begin{array}{cc} \left(\tau_{t,r_{1}}^{s}+\tau_{t,r_{1}}^{p}\right)-\left(\tau_{t,r_{2}}^{s}+\tau^{HO}\right) & \leq My_{t,r_{1},r_{2}}^{\prime } \end{array}$$

(11b) $$\begin{array}{cc} y_{t,r_{1},r_{2}} & \leq x_{t,r_{1}} \end{array}$$

(11c) $$\begin{array}{cc} y_{t,r_{1},r_{2}} & \leq x_{t,r_{2}} \end{array}$$

(11d) $$\begin{array}{cc} y_{t,r_{1},r_{2}} & \leq y_{t,r_{1},r_{2}}^{\prime } \end{array}$$

(11e) $$\begin{array}{cc} x_{t,r_{1}}+x_{t,r_{2}}+y_{t,r_{1},r_{2}}^{\prime }-2 & \leq y_{t,r_{1},r_{2}} \end{array}$$

(11f) $$\begin{array}{cc} \left(\tau_{t,r_{2}}^{s}+\tau^{HO}\right) & -\left(\tau_{t,r_{1}}^{s}+\tau_{t,r_{1}}^{p}\right)=M(1-y_{t,r_{1},r_{2}}) \end{array}$$

$$\forall t\in T,\;\;\;\;r_{1},r_{2}\in R,\;\;\;\;\delta_{t,r_{1}}^{TW}<\;\delta_{t,r_{2}}^{TW}\;\;\mathrm{for}\;\;(11a)-(11f)$$

(11g) $$\begin{array}{c} y_{t,r_{1},r_{2}}+x_{t,r}\leq 1 \end{array}$$

$$\forall t\in T,\;\;\;r_{1},r,r_{2}\in R,\;\;\;\begin{array}{l} \gamma_{t,r_{1}}^{TW}<\gamma_{t,r}^{TW}<\gamma_{t,r_{2}}^{TW},\;\;\;\delta_{t,r_{1}}^{TW}<\delta_{t,r}^{TW}<\delta_{t,r_{2}}^{TW} \end{array}\;\;\;\;\;\mathrm{for}\;(11g)$$

The constraints in Equation (5) bind target–radar assignment indicators to a single variable with an OR operator.

The constraints in Equation (6) represent the lower bound of the tracking duration for each target–radar pair if the corresponding binary indicator variable $x_{t,r}$ equals 1. *M* in the equations is a very large positive number and used to effectively activate the constraint only when the variables multiplied to this *M* take zero [32]. Briefly, $\tau^{p,min}x_{t,r}\leq \tau_{t,r}^{p}$ if $x_{t,r}=1$, otherwise $\tau_{t,r}^{p}$ becomes 0.

The constraints in Equation (7) ensure the lower and upper bounds of the start and end time for each target–radar pair; the assignment should be inside the corresponding time window.

Let us call the constraints in Equation (8) the occupying constraints. For target–radar pair (t,r), $\theta_{t,r,i}$ indicates whether an assignment exists or not in Interval *i*. Briefly, $\theta_{t,r,i}$ equals 1 if the following conditions are met simultaneously: $y_{t,r}=1$, $\tau_{t,r}^{s}\leq \delta_{i}^{Int}$, and $\gamma_{i}^{Int}\leq \tau_{t,r}^{s}+\tau_{t,r}^{p}$.

The constraints in Equation (9) limit the maximum number of radars used for tracking a single target to two; two radars are assigned when the handover occurs, otherwise a single radar tracks the target. Simply, the handover of a single target will only occur between two radars. The constraints in Equation (10) limit the capability of simultaneous tracking for each radar.

The constraints in Equation (11) are for the handover. To decide the handover indicator variable $y_{t,r_{1},r_{2}}$, we define the support variable $y_{t,r_{1},r_{2}}^{\prime }$ as in Equation (11a); $y_{t,r_{1},r_{2}}^{\prime }$ is 1 if the end time of Radar $r_{1}$ tracking Target *t* is equal to or higher than the sum of start time and handover duration for Radar $r_{2}$. The reason for using the support variable is that both target–radar pairs $\left(t,r_{1}\right)$ and $\left(t,r_{2}\right)$ must be assigned the schedule simultaneously as well as satisfying the handover time constraint (Equation (11b)–(11f)). The constraints in Equation (11g) ensure that radars with duplicating and smaller time windows are excluded from the assignment.

## 4. Results and Discussion

In this section, computational results for test instances of the multiple radar resource scheduling problem described in Section 3 are reported. The optimization for the mixed-integer linear problem was solved by Gurobi 8.0.1 based on Python 3.6.2 and the optimization for the heuristic problem was solved with the same Python environment. The computation was conducted by a desktop with an Intel Core^TM^ i7-6700K, 4.00 GHz CPU and 16 GB of RAM.

**Figure 4:**
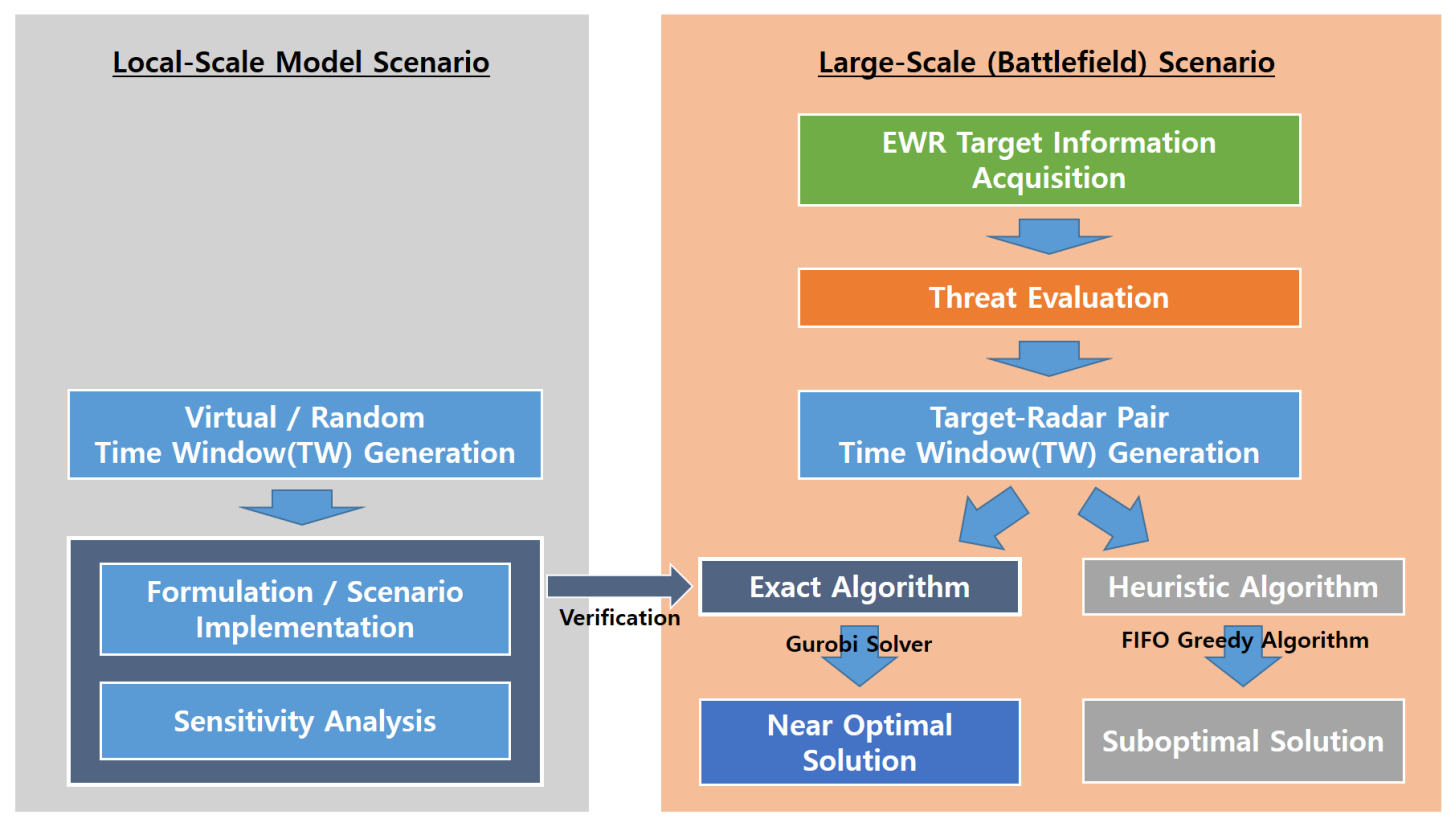
Overview of the numerical experiment.

Two experimental models were tested to verify the effectiveness of the algorithm, as shown in Figure 4. The first scenario verified the effectiveness of the exact algorithm using a local-scale model for easy parameter modification. The second scenario verified the practical applicability of the algorithm by introducing two approaches for the large-scale (battlefield) scenario, which considered far more virtual targets and radars than were used in the local-scale model.

### 4.1. Local Scale Scenario Experiment

#### 4.1.1. Algorithm Verification

**Figure 5:**
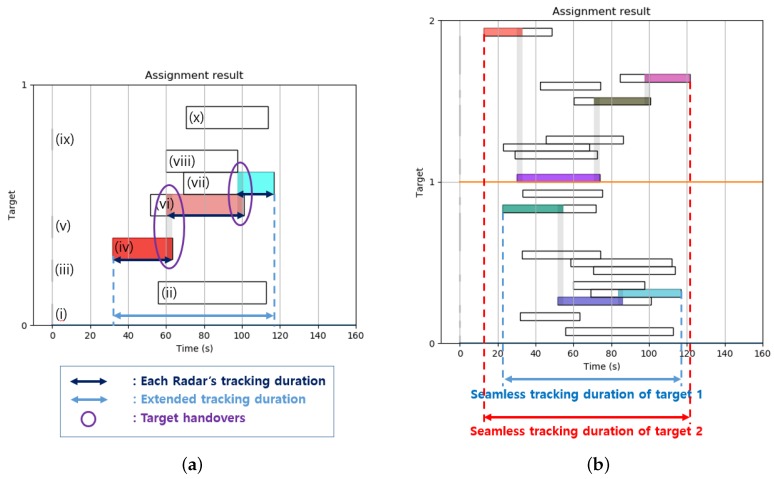
Optimal scheduling assignment results obtained using simple local-scale model. (**a**) One target and 10 radars. (**b**) Two targets and 20 radars.

We verified the algorithm using a local-scale model to confirm that the objective function and constraints work well. The local-scale model allowed us to arbitrarily set the number of targets and radars. Among the four objective function terms, the first one “target importance” was assumed to be constant for this simple simulation. To check for changes according to the number of available radars, we first tested two different cases of 10 radars for a single target, and 20 radars for two targets. Figure 5a shows the tracking results for a single target using 10 radars with different time windows. The size of each time window was set to be randomly generated within a maximum of 60 s and a minimum of 30 s. Other parameters used here are shown in Table 3.

As can be seen in Figure 5a, among the 10 radars, six radars were involved in target tracking, because the probability that each radar can participate in tracking the target was set as equal to or less than 60%. Two target handovers occurred in the relevant sections of (iv)–(vi) at 60 s and (vi)–(vii) at 97 s, and the time window was selected to keep track of the target for as long as possible while maintaining the minimum takeover time as designed in the objective function. What is unique here is that, although Time Window (ii) is longer than any of the others, the solver assigned targets to Time Windows (vi) and (vii) to track the target as long as possible and at the same time to meet the constraint of the minimum tracking assignment time. On top of the conditions given in the results shown in Figure 5a, Figure 5b shows the results of the tracking assignment of 20 radars for two targets, as well as results for adding one more target and 10 more radars. These results also show that radar resources were well assigned to reflect the designed objective function and the constraints, such that the first target required two handovers and the second target required three handovers to achieve maximum tracking of each target.

Thus far, we verified two of the four terms of the objective function in Equation (4), namely maximization of target tracking time and minimization of the number of target handovers, as well as the constraints, are working well. In the above test model, since the target number was set too small, the third term of the objective function, that is, the test result required to maximize the number of targets to track, could not be confirmed. Therefore, in the following experiment, to see how all the terms of the objective function can be demonstrated, we increased the number of targets and limited the number of radar. This involved one of the key parameters in Table 1, $n^{\mathrm{capa}}$, the simultaneous tracking capability of each radar.

**Figure 6:**
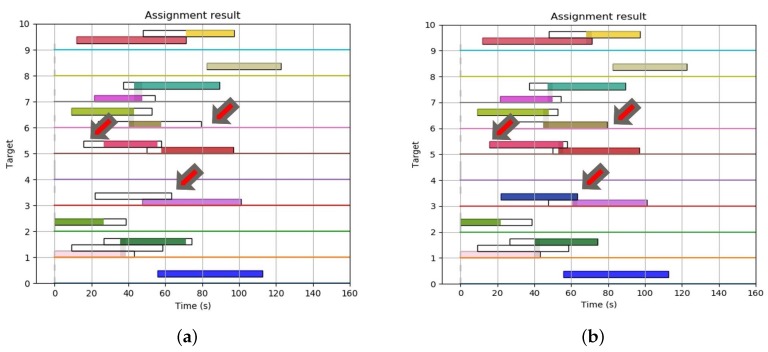
Optimal scheduling assignment result for 10 targets and three radars with different simultaneous tracking capability. (**a**) When the radars can track two targets simultaneously ($n^{\mathrm{capa}}=2$). (**b**) When the radars can track three targets simultaneously ($n^{\mathrm{capa}}=3$).

Using the parameters in Table 3, Figure 6a depicts the optimal scheduling results for tracking 10 targets with three radars. For each target, depending on the detection probability of 60% mentioned above, we can confirm that 1–3 radars were assigned to all targets except for the fourth target, which could not be detected and tracked in this simulation condition. Figure 6b depicts what happens when the radar’s simultaneous tracking capability ($n^{\mathrm{capa}}$) is adjusted to 3. The most noticeable thing is that the tracking durations of the fourth, sixth and seventh targets increased dramatically, as shown by the red colored arrows in Figure 6. Especially, it was possible to track the fourth target only in the time window of the first radar, as shown in Figure 6a; however, as $n^{\mathrm{capa}}$ increased, the target could be tracked in all available time window sections of the first and second radar, as shown in Figure 6b. In addition, considering the number of targets being tracked at 70 s in Figure 6, it can be seen in Figure 6b that seven targets could be tracked, while six targets could be tracked in Figure 6a. Thus, although there is a difference in degree, as the tracking ability improved, the tracking time for the entire target generally improved, as shown in Table 4.

This is the result, for certain targets, of slightly increasing or decreasing that tracking time according to the terms of the objective function in Equation (4) and the constraint “simultaneous tracking capability of radar ($n^{\mathrm{capa}}$)”, written in Equation (10). The values in this table are the time taken from the moment the target was first detected by one radar to the moment it was lost after being handed over to another radar. One more noticeable point in Figure 6 is that, in the case of the 10th target, the minimum time required for the handover was not met because of the limitation of the simultaneous tracking capability, as shown in Figure 6a, while the target handover can be seen to have been smoothly accomplished in Figure 6b due to the increase in the tracking duration time of the third radar.

**Figure 7:**
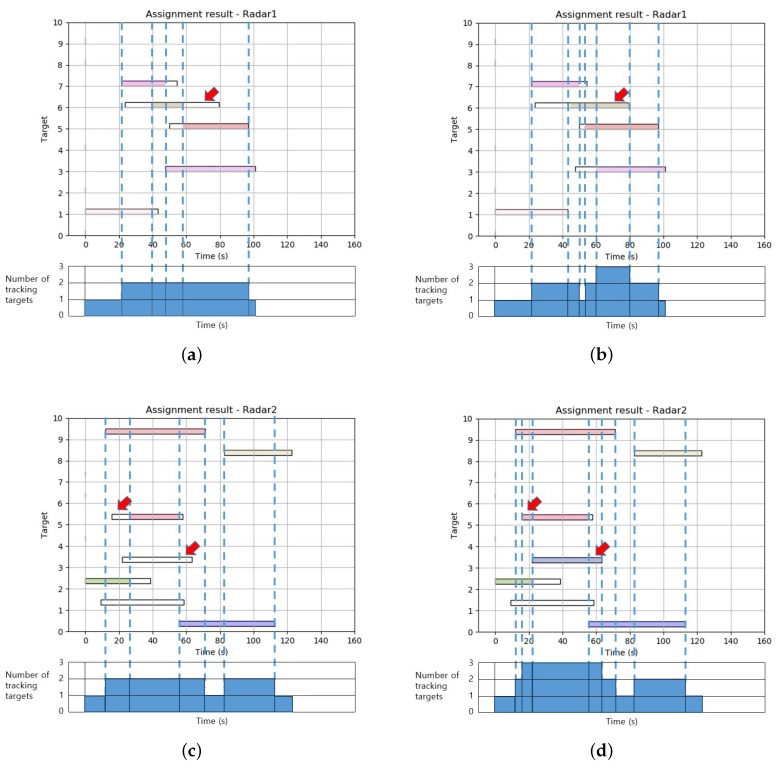
Optimal scheduling assignment result of each radar site according to the change of simultaneous tracking capability. (**a**) Assignment result of first radar when $n^{\mathrm{capa}}=2$. (**b**) Assignment result of first radar when $n^{\mathrm{capa}}=3$. (**c**) Assignment result of second radar when $n^{\mathrm{capa}}=2$. (**d**) Assignment result of second radar when $n^{\mathrm{capa}}=3$.

Figure 7 shows how the tracking of a target actually changed in the time window of each radar as the simultaneous tracking capability ($n^{\mathrm{capa}}$) changed.

In Figure 7a,b, which are the assignment results for Radar 1, it is confirmed that the number of targets to be tracked throughout the whole planning horizon did not exceed a maximum of 2, in Figure 7a, and 3, in Figure 7b. Looking more closely at Figure 7b, we can observe that three targets were being tracked at the same time only between about 60 and 80 s as the tracking duration time of the seventh target expanded. Similarly, in Figure 7c,d, the tracking duration for the fourth and sixth targets expanded with increased simultaneous tracking capability, and thus three targets were being tracked simultaneously between about 15 and 60 s.

#### 4.1.2. Parameter Sensitivity Analysis

As explained in the previous case, the objective value of the optimal scheduling problem depends on changes in the value of a particular parameter. Therefore, we performed a sensitivity analysis to determine how the parameters affect the outcome of the objective function. Three parameters were determined to affect the results. Sensitivity analysis was performed by fixing the remaining parameters while adjusting one target parameter, as shown in Table 5. The target parameter for the first sensitivity analysis was the simultaneous tracking capability ($n^{\mathrm{capa}}$) of the radar, as shown in Table 5.

**Figure 8:**
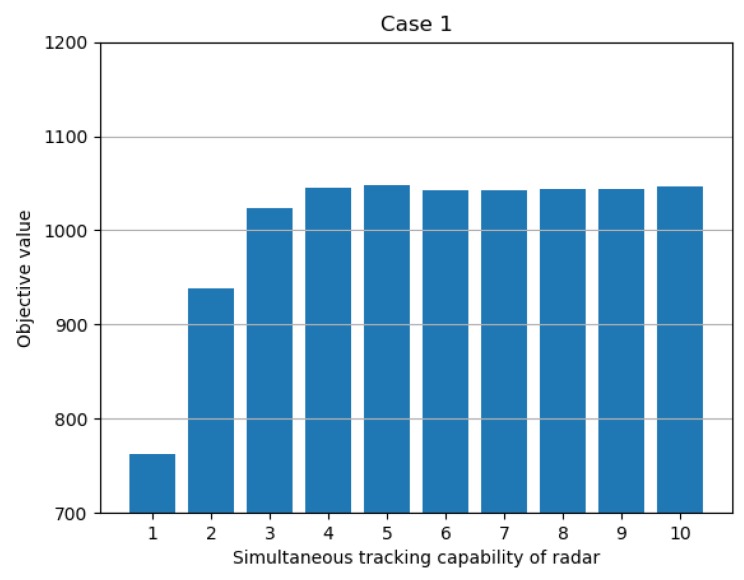
Objective value with respect to number of simultaneous tracking capability.

Figure 8 shows how the results varied with the simultaneous tracking capability. As shown in Table 4, the result of the objective function initially increased when $n^{\mathrm{capa}}$ increased. However, it can be seen that, after a certain level, the result of the objective function was not significantly affected. This tendency was only the result of a given condition, and, when the condition changed, a point that was not affected by the change of $n^{\mathrm{capa}}$ could be changed. Specifically, if the simultaneous tracking capability of the radar covered the number of targets, the influence of $n^{\mathrm{capa}}$ would be insignificant. If the number of targets to be tracked were greater than the simultaneous tracking capability of the radar, when the value of $n^{\mathrm{capa}}$ is high, the objective value would also rise, as shown in Figure 8, until the radars can cover all the targets.

**Figure 9:**
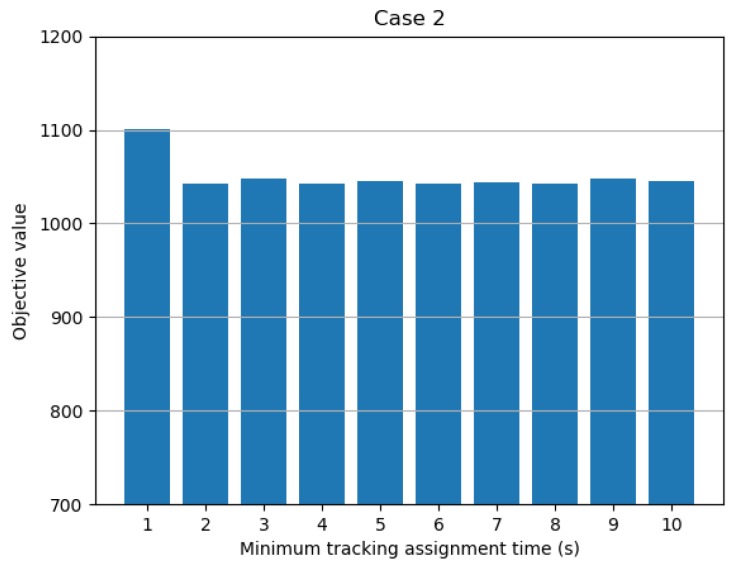
Objective value with respect to minimum tracking assignment time.

The second parameter that affects the value of the objective function is the minimum tracking assignment time. This parameter is the minimum time required for a radar to track a target, and physically refers to the time it takes for the radar filter system to stabilize the target tracking. Figure 9 shows the objective value according to the change of the minimum tracking assignment time. As can be seen in the figure, smaller minimum tracking assignment times led to higher levels of assignment, but the assignment was not affected after a certain level of minimum tracking assignment time.

**Figure 10:**
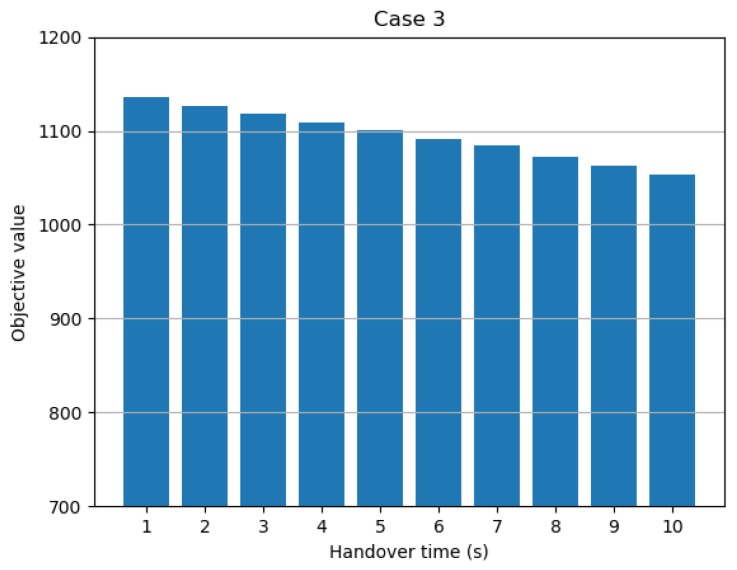
Objective value with respect to handover time.

The third parameter for the sensitivity analysis was the time required to hand over the target between networked radars. In fact, this is a simulation parameter, and in a real environment it is very likely to be affected by the physical state of the network and filtering system. However, since it is a parameter that has an important influence on the simulation results, it was selected as a parameter in the sensitivity analysis to grasp its influence and overall tendency. As shown in Figure 10, the objective value decreased linearly as the handover time increased. This result shows that longer handover times led to less efficient overall target tracking. Therefore, a shorter handover time is better. In other words, the network system actually should be constructed so as to minimize the time required for target transmission, as well as the time required for convergence between a transmitted target and a self-detected target.

### 4.2. Battlefield Scenario Experiment

**Figure 11:**
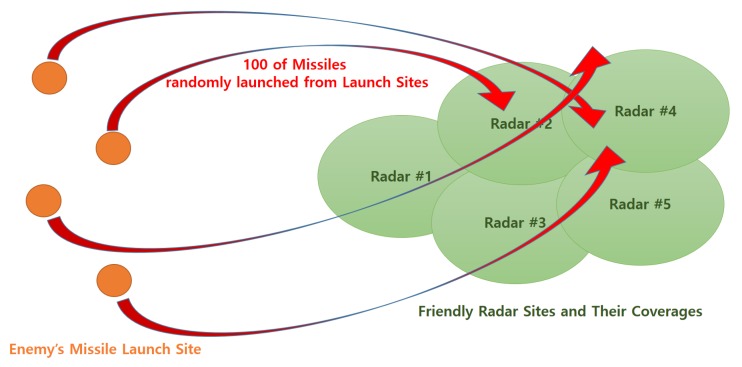
Conceptual diagram for the battlefield scenario.

In this experiment, scheduling optimization was performed assuming a situation in which 100 enemy ballistic missiles of four different types from four different launch sites flocked toward five friendly radar sites distributed appropriately. This is a much worse situation than that of local-scale model problem. Through this experiment, we verified the effectiveness and practical applicability of the optimal scheduling technique that employs the seamless handover method proposed in this study. Table 6 and Figure 11 show the parameters and conceptual diagram for this experiment, respectively. Compared to the local-scale model, the optimization planning horizon was increased to 1000 s and the number of targets and radars increased to 100 and 5, respectively. The most important parameter—the simultaneous tracking capability—was increased to 20 so that five radars could cover all the 100 targets.

One of the most different aspects compared to the local-scale model is the importance of target, which is the first term of Equation (4). It is reflected in the objective function for this scenario unlike the previous experiment. The problem was solved with the assumption that the importance of target is uniform in the previous experiment. However, in this scenario, the target distance from radar and the response time available for the target were taken into consideration, as written in Equation (1), as in a real situation. Another difference related to the time window creation. In the local-scale model, a random function was used to generate a time window between arbitrary times selected by the user. However, in this experiment, it was assumed that the early warning radar provides the trajectory information of the ballistic missiles, so that the time windows could be created for radars located in various regions.

#### 4.2.1. Weights of Objective Function Sensitivity Analysis

The objective function used in Equation (2) can be said to have some form of weighted sum. To check the dominance of each term of the objective function, the optimum value of individual objective was checked, as shown in Table 7.

As shown in Table 7, the first term, the priority of the target, was the dominant term that had the greatest influence on the objective function value. The third, the maximization number of tracked target, was found to have very little effect compared to the others. The fourth term, the minimization of the number of handover, is not included in this table because it acted as a penalty term. To analyze properly, the objective function needed to be normalized. In this study, considering the penalty terms, instead of dividing the objective by those optimum values, the most influential objective’s coefficient ($c_{1}$) was set to 1 and the remaining coefficients were normalized accordingly.

**Figure 12:**
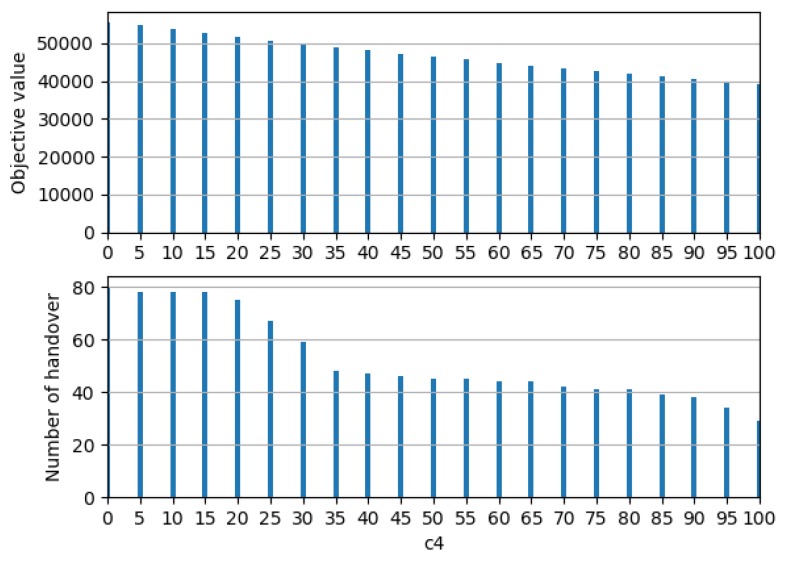
Sensitivity with respect to the penalty term.

Based on the coefficients determined above, a sensitivity analysis was conducted according to the penalty term, the minimization of the number of handover, as shown in Figure 12. It is trivial that the objective function value decreased with increasing $c_{4}$. One interesting point is that the number of handover decreased step-wise. These results indicate that, if $c_{4}$ is smaller than necessary, the overall objective function value can be high, but there are many unnecessary handovers. Therefore, choosing $c_{4}$ at which it starts to no longer decrease is the best decision to maximize the objective function value and reduce the number of handovers. Thus, $c_{4}$ of 35 was chosen for this case.

#### 4.2.2. Complexity Analysis

In general, the Mixed Integer Linear Program (MILP) problem is known as NP-hard or NP-complete problem. It is also known that NP-hard problem has exponential computational complexity [33]. Therefore, in this section, we look at how the complexity changes according to the parameters that affect the computational complexity, and to what extent we can use this algorithm using MILP formulation. To achieve that, we confirmed how the complexity appeared according to the number of targets, the number of radars, and the number of targets that can be simultaneously detected by radars ($n^{\mathrm{capa}}$).

**Figure 13:**
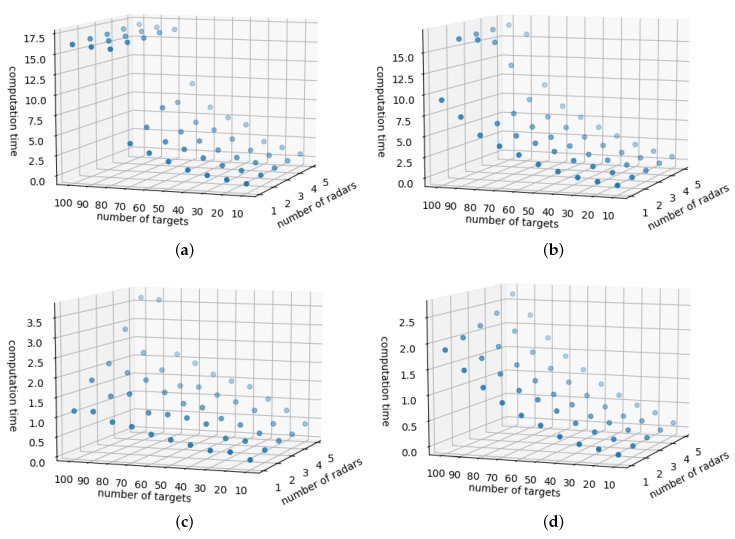
MILP formulation complexity as of computation time according to the change of simultaneous tracking capability. (**a**) Computation time when $n^{\mathrm{capa}}=10$. (**b**) Computation time when $n^{\mathrm{capa}}=15$. (**c**) Computation time when $n^{\mathrm{capa}}=20$. (**d**) Computation time when $n^{\mathrm{capa}}=25$.

First, the most prominent in Figure 13 is the exponentially increasing computation time, as previously predicted. The most important parameter for analyzing here is the $n^{\mathrm{capa}}$. The greater is the radar’s ability to track simultaneously, the shorter id the calculation time due to the less load, in which case a gentle exponential curve is drawn. On the contrary, when the radar’s simultaneous tracking capability is low, the calculation time explodes, and it is confirmed that the calculation is very slow in an overload situation exceeding a certain number of targets. On the other hand, the calculation time increase is more sensitive to the number of targets than to the number of radars. Based on this, it can be concluded that, when designing a radar network, it is very advantageous for the target and sensor assignment of multiple targets if we increase the simultaneous tracking capability of each radar. Based on these data, we can also establish the algorithm re-planning cycle that is envisioned in the future.

#### 4.2.3. Numerical Simulations for Comparison

In this scenario, the proposed MILP solution was compared with a heuristic technique, termed the First-in First-out (FIFO) greedy algorithm (Appendix A). This greedy heuristic is an extension of a sequential greedy algorithm for assignment [34] to take into account the handover requirement. Note that the greedy scheme can be a good reference algorithm as it works very well in many domains and also guarantees some optimality gap when the objective function satisfies certain conditions [34,35]. Detailed theoretical analysis of the greedy heuristic is omitted as it is out of the focus of this paper.

The overall procedure is well described in Figure 4.

**Figure 14:**
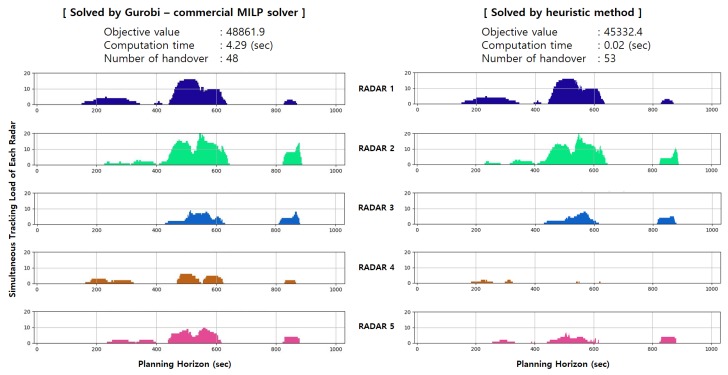
Simulation result comparison in the sense of the Number of simultaneously tracked target for each radar.

Figure 14 shows the number of targets being simultaneously tracked by each radar over the planning horizon for the exact and heuristic algorithms. As can be seen in the figure, the simultaneous tracking load of each radar clearly increased between 400 and 650 s because targets were the most frequent and concentrated at that time. In terms of an objective value, the result of the proposed formulation solved by Gurobi commercial MILP solver returned 48861.9, while the heuristic algorithm gave a value of 45,332.4, an approximately 8% difference in performance. This was noticeably exhibited mainly in the simultaneous tracking loads of Radars 4 and 5, as shown in Figure 14. The reason for this is that the heuristic approach to solving this problem is to maximize the time that each radar tracks in a greedy manner. This phenomenon is explained by the local optima convergence, which is a typical disadvantage of the heuristic approach, and therefore shows an assignment result that is not properly distributed. Meanwhile, 48 handovers took place between the radars in the case of exact algorithm while 53 handovers occurred in the case of heuristic algorithm. When we compared performance with these results, we considered two main things: the number of handovers that act as the the penalty function in the objective function and the objective value obtained. The simulation results are more than simply comparing the objective values and having a low number of handovers. The absence of unnecessary handovers is much more advantageous in terms of radar operation. Although there are differences in the number of handovers depending on how to solve the problem, the target can be tracked for a much longer period of time than in the case without handovers between radars, and the resulting time margin would provide valuable time for the preparation of the next battle for each interceptor. The computation time of exact algorithm case was approximately 4.27 s longer because the solution using the Gurobi solver investigated as many cases as possible to find the optimal solution.

## 5. Conclusions

Using a local-scale model and real world example, we experimented with optimal scheduling of multiple radars for multiple targets and derived appropriate results. Simulation results show that the objective function of the proposed formulation is valid and effective for the real world situation by using target handover. The experiment results show that the proposed exact algorithm solved using Gurobi exhibited better results than that of the heuristic method in terms of performance, number of handovers and tracking load for entire systems. This paper opens the possibility of solving the problem of seamless multi-target tracking of multiple radar network against a large number of missile attacks. The results are especially helpful in preventing situations in which radars with limited detection area are unnecessarily tracking multiple targets at the same time. The resulting margin of tracking capability will increase the survivability in such situations. For future work, first, we will try to find adaptive parameters that may not be confined to a specific situation for each coefficient of objectives constituting the objective function. Second, we will expand and connect this sensor assignment problem into the weapon target assignment problem for an anti-air defense system which is composed of multiple radars and multiple interceptor systems. Third, we will continue to study the methodology to apply the Reinforcement Learning (RL) technique to this problem.

## Figures and Tables

**Table 1:** List of parameters.

Notation	Physical Meaning
$\gamma_{t,r}^{TW}$	Start time of time window
$\delta_{t,r}^{TW}$	End time of time window
$\gamma_{i}^{Int}$	Start time of Interval *i*
$\delta_{i}^{Int}$	End time of Interval *i*
$\tau^{p,min}$	Minimum tracking assignment time
$\tau^{HO}$	Target handover time
$\omega_{t}$	Target priority(importance of target)
$n^{R}$	Number of radars
$n^{T}$	Number of targets
$n^{\mathrm{capa}}$	Simultaneous tracking capability of each radar

**Table 2:** List of decision variables.

Notation	Value	Physical Meaning
$\tau_{t,r}^{s}$	$\in R^{+}$	Start time of tracking
$\tau_{t,r}^{p}$	$\in R^{+}$	Tracking duration time
$x_{t}$	∈{0,1}	Whether Target *t* is being allocated (tracked) or not
$x_{t,r}$	∈{0,1}	Whether Radar *r* tracks the target *t* or not
$y_{t,r_{1},r_{2}}$	∈{0,1}	Whether Radar $r_{1}$ and $r_{2}$ handover the target *t* or not
$y_{t,r_{1},r_{2}}^{\prime }$	∈{0,1}	Support variable for $y_{t,r_{1},r_{2}}$
$\theta_{t,r,i}$	∈{0,1}	Whether Radar *r* tracks the Target *t* in interval *i* or not

**Table 3:** Parameter set for local-scale model simulation.

Planning horizon	160 s
Minimum tracking assignment time	7 s
Target handover time	3 s
Simultaneous tracking capability of each radar $\left(n^{\mathrm{capa}}\right)$	2

**Table 4:** Tracking duration time according to simultaneous tracking capability.

Target Number	Total Tracking Duration (s)		
	$n^{\mathrm{capa}}=2$	$n^{\mathrm{capa}}=3$	Increments
Target 1	56.8	56.8	0
Target 2	71.1	74.3	+3.2
Target 3	26.7	21.8	−4.9
Target 4	53.4	79.2	+25.8
Target 5	0	0	0
Target 6	70.2	81	+10.8
Target 7	48.8	70.1	+21.3
Target 8	67.9	67.9	0
Target 9	40.2	40.2	0
Target 10	85.4	85.4	0

**Table 5:** Parameter setting for sensitivity analysis.

Parameters	Case 1	Case 2	Case 3
Simultaneous tracking capability	1 to 10	5	5
Minimum tracking assignment time	10	1 to 10	10
Handover time	10	10	1 to 10

**Table 6:** Parameter set for battlefield scenario experiment.

Number of target $\left(n^{T}\right)$	100
Number of radar $\left(n^{R}\right)$	5
Planning horizon	1000 s
Minimum tracking assignment time	7 s
Target handover time	3 s
Simultaneous tracking capability of each radar $\left(n^{\mathrm{capa}}\right)$	20

**Table 7:** Solution ranges of each term of objective function and proposed coefficient setting.

Term	Opt. of Ind. Objective	Coeff. Value for Normalization
Target priority	18,610.8	$c_{1}=1$
Maximization of tracking time	7621.6	$c_{2}=2.44$
Maximization of the number of tracked target	98.0	$c_{3}=189.9$

## Appendix A. Pseudo-Code of FIFO Greedy Algorithm

**Algorithm A1** FIFO greedy algorithm
Input: Target information, Time windows information, Radar tracking capability
Output: Target tracking schedules for each radar
1: **procedure** Schedule FIFO 2: *TW data* ← array with start and end time elements 3: ▹ TW data: Set of time windows generated based on targets’ movement and radar’s location 4: t← number of target 5: r← number of radar 6: $\tau \leftarrow \tau_{o},\tau_{f}$ 7: v← 0,1 ▹ v: Indicator that tells whether it is the release time(0) or the due time(1) of TW 8: **for** t,r,τ,v in *TW data* **do** 9: t,r,τ,v←ϕ 10: **for** each *TW data* in the scenario **do** 11: [t,r]← target and radar pair of TW 12: $\tau_{o}\leftarrow$ release time of TW 13: v← 0 14: *TW data* ←*TW data* $⋃(t,r,\tau_{0},v$) 15: $\tau_{f}\leftarrow$ due time of TW 16: v← 1 17: *TW data* ←*TW data* $⋃(t,r,\tau_{f},v$) 18: **end for** 19: **end for** 21: Sort *TW data* based on ascending time order 23: **for** $t,r,\tau_{o},v$ in *TW data* **do** 24: **if** *v* is 0 **then** ▹v=0: τ is release time 25: **if** target t is not on tracking **then** 26: $\tau_{o},\tau_{f}\leftarrow TW_{o}\left[t,r\right],TW_{f}\left[t,r\right]$ 27: **if** radar’s resource available from $\tau_{o}$ to $\tau_{f}$ **then** 28: Assign Radar *r* to Target *t* between $\tau_{o}$ and $\tau_{f}$ 29: **end if** 30: **else** $r_{last}\leftarrow$ last radar on target 31: **if** Target *t* is on tracking but not reached the end of $TW_{f}\left[t,r\right]$ **then** 32: Do not handover yet 33: **if** the correlated TW of the two radars satisfies enough handover time **then** 34: Perform handover 35: **else** Do not handover 36: **end if** 37: **end if** 38: **end if** 39: **else** ▹v=1: τ is due time 40: Terminate Radar *r*’s tracking 41: **end if** 42: **end for** 43: **end procedure**
